# Shared Genetic Links Between Sleep, Neurodevelopmental and Neuropsychiatric Conditions: A Genome‐Wide and Pathway‐Based Polygenic Score Analysis

**DOI:** 10.1111/gbb.70011

**Published:** 2024-12-26

**Authors:** Laura Fahey, Lorna M. Lopez

**Affiliations:** ^1^ Department of Biology Maynooth University Maynooth County Kildare Ireland

## Abstract

Genetic correlations have been reported between chronotype and both autism (AUT) and schizophrenia (SCZ), as well as between insomnia and attention‐deficit/hyperactivity disorder (ADHD), bipolar disorder (BP), schizophrenia (SCZ) and major depression (MDD). Our study aimed to investigate these shared genetic variations using genome‐wide and pathway‐based polygenic score analyses. We computed polygenic scores using summary statistics from genome‐wide association studies (GWAS) of ADHD (*N* = 225,534), AUT (*N* = 46,350), BP (*N* = 353,899), MDD (*N* = 500,199) and SCZ (*N* = 160,779). We tested their performance in predicting chronotype (*N* = 409,630) and insomnia (*N* = 239,918) status of UK Biobank participants. For pathway‐based polygenic scores, we restricted genetic variation to SNPs that mapped to genes within 1377 Reactome pathways. Genome‐wide polygenic scores for AUT, BP, MDD and SCZ were associated with an evening chronotype (*p* < 2.2 × 10^−16^, *p* = 4.8 × 10^−3^, *p* = 8.07 × 10^−4^ and *p* < 2.2 × 10^−16^, respectively). Polygenic scores for ADHD, AUT, BP, MDD SCZ were associated with insomnia (*p* < 2.2 × 10^−16^, *p* = 2.93 × 10^−3^, *p* = 2.9 × 10^−7^, *p* < 2.2 × 10^−16^ and *p* = 8.86 × 10^−3^, respectively). While pathway‐based polygenic score analysis identified the KEAP1‐NRF2 (*p* = 1.29 × 10^−8^) and mRNA Splicing‐Minor Pathways (*p* = 1.52 × 10^−8^) as enriched for genetic variation overlapping between chronotype and BP, the majority of tested pathways yielded null findings, suggesting that specific shared genetic mechanisms between sleep‐related phenotypes and neurodevelopmental/psychiatric conditions (NDPC) may be limited to a subset of pathways. Colocalisation analysis identified BP‐associated SNPs in *CUL3* and *SF3B1* as being linked to changes in their expression. Our results strengthen evidence for shared genetic variation between NDPC and sleep‐related phenotypes. We identify the KEAP1‐NRF2 and mRNA Splicing‐Minor Pathways as potentially mediating the disrupted circadian rhythm phenotype of BP.

## Graphical abstract

This study investigates shared genetic influences that may contribute to the circadian rhythm disruption and sleep issues in neurodevelopmental and neuropsychiatric conditions (NDPCs). Our findings reveal that polygenic scores (PGSs) for autism, schizophrenia and bipolar disorder are associated with an evening chronotype, while polygenic scores for attention‐deficit/hyperactivity disorder (ADHD), autism (AUT), schizophrenia (SCZ) and bipolar disorder (BP) are associated with insomnia, indicating shared genetic variation between these phenotype pairs. Pathway‐based polygenic score analysis identified the enrichment of shared genetic variation between chronotype and BP in the NRF2‐KEAP1 and mRNA splicing minor pathways.

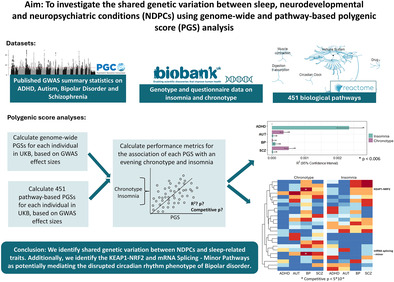

## 1 Introduction

Sleep disturbances have high prevalence across almost all neuropsychiatric and neurodevelopmental conditions. They are considered a core symptom in the diagnostic criteria for bipolar disorder (BP) [1], present in up to 80% of individuals with schizophrenia (SCZ) [2] and autism spectrum disorder (autism, AUT) [3, 4] and observed in up to 50% of those with attention‐deficit/hyperactivity disorder (ADHD) [5]. It is understandable that the symptoms of neurodevelopmental/psychiatric conditions (NDPCs) would lead to sleep disruption, and that sleep disruption would worsen the symptoms of NDPCs. However, sleep disturbances are also associated with the development of NDPCs, as indicated by findings that sleep disturbances often predate onset of major depressive disorder and BP [6]. Moreover, mouse models with mutations in circadian clock genes, which regulate sleep timing and other biological processes through transcription–translational feedback loops, display symptoms reminiscent of human NDPCs [7]. This led to the hypothesis that circadian disruption is a common underlying mechanism, contributing to both the development of neuropsychiatric conditions and sleep disruption [7].

**FIGURE 1:**
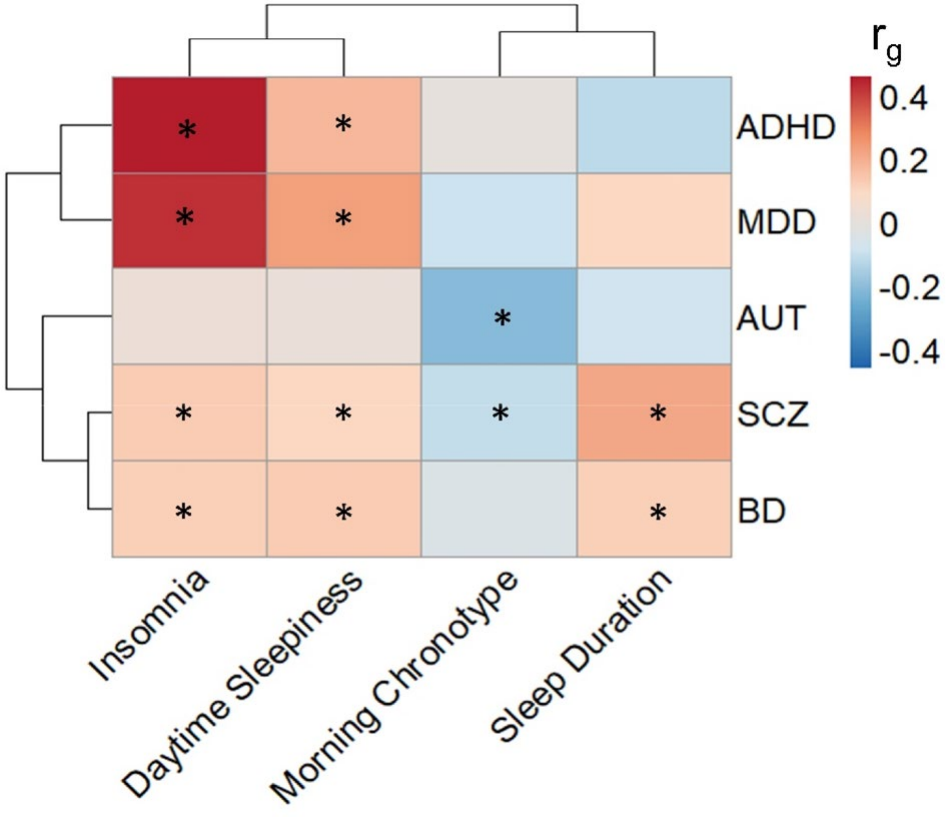
Published genetic correlations (*r*_g_) between pairs of neuropsychiatric or neurodevelopmental and sleep phenotypes. Heatmap cells marked with an asterisk (*) indicate that they were reported as statistically significant in the original publications, after multiple testing correction. The original publications include the latest and largest genome‐wide association studies of Autism (AUT [15]), morning chronotype [25], attention‐deficit hyperactivity disorder (ADHD [16]), bipolar disorder (BP; [18]), major depressive disorder (MDD; Howard et al. 2019), sleep duration [40] and daytime sleepiness [41]. Rows and columns are clustered based on *r*_g_ similarity, as illustrated by the dendrograms.

Genomic studies also support common mechanisms underlying NDPC and sleep traits. Genome‐wide correlations in genome‐wide association studies (GWAS) identified single nucleotide polymorphism (SNP) effects have been reported between many sleep‐related traits and NDPCs using the linkage disequilibrium score regression tool (Figure 1). Chronotype, representing an individual's behavioural expression of their circadian clock, is often used as a proxy phenotype for circadian timing [8]. In modern society, having a morning chronotype is considered advantageous due to its alignment with early school and work start times. Conversely, evening chronotypes have being associated with numerous adverse psychological outcomes [9]. GWAS identified SNP effects associated with a morning chronotype have been reported to be negatively correlated with many NDPC phenotypes. Whereas, debilitating phenotypes, such as insomnia and daytime sleepiness, exhibit positive genetic correlations with NDPCs (Figure 1).

Polygenic score (PGS) analysis can also be used to assess genetic correlations between phenotypes using independent discovery and target datasets. If a PGS computed using effect sizes from a GWAS of a particular phenotype in the discovery dataset can predict a different phenotype in an independent target cohort, it indicates shared genetic variation between the two phenotypes [10]. PGSs for ADHD and depression have been reported to positively correlate with initiating and maintaining sleep and excessive somnolence in children. The same study found a PGS for anxiety disorder to correlate with nightmares in children [11].

Exploring the genes, biological pathways and tissue types impacted by the shared genetic variation between sleep and NDPCs has been a relatively unexplored area. A gene‐based cross‐trait meta‐analysis identified 44 genes common to both insomnia and ADHD that were enriched for functionality in synaptic‐related pathways [12]. Another study identified 149 loci shared between psychiatric disorders (SCZ, BP and depression) and sleep traits (chronotype and sleep duration). Overall, 49 lead SNPs at these loci were found to act as eQTLs for 115 genes across various brain tissues including the basal ganglia, cortex, hippocampus and cerebellum [13].

The objectives of this study were two‐fold. To investigate shared genetic variation, our first objective was to assess whether genome‐wide polygenic scores for ADHD, AUT, BP, major depressive disorder (MDD) and SCZ, and could predict insomnia and chronotype status of participants in the UK Biobank (UKB). Our second objective was to test for enrichment of shared genetic variation in different biological pathway using pathway‐based polygenic score analysis. We report that polygenic scores for AUT, BP, MDD and SCZ were associated with chronotype status in UKB and polygenic scores for all five NDPC phenotypes were associated with insomnia in UKB. BP polygenic scores, restricted to genetic variation in the KEAP1‐NRF2 and mRNA splicing‐minor Reactome pathways, predicted chronotype in UKB, over and above the genome‐wide genetic background signal. To gain further biological insights, we investigated expression quantitative trait loci (eQTL) data and found eQTLs associated with changes in the expression of genes within these pathways to colocalise with BP associated SNPs. However, we did not find these gene‐sets to be enriched in genes differentially expressed in the dorsolateral prefrontal cortex of BP cases.

## 2 Datasets and Methods

### 2.1 Target Data for Polygenic Score Analysis—UK Biobank

Two UKB phenotypes were used for this study—chronotype (data‐field 1180) and insomnia (data‐field 1200). Both were collected through participant questionnaires administered on touchscreen computers at UKB assessment centres. We encoded chronotype as an eveningness phenotype and insomnia as a binary phenotype (Table S1). We removed samples based on relatedness using the UKB supplied relatedness file, which lists pairs of individuals related up to a third degree. One individual from each pair was removed without removing samples from pairs where a sample had already been removed. Further exclusions were made based on samples that did not belong to the UK Biobank's White British ancestry subset (Data‐Field 22,006), which combines self‐identified ethnicity with principal component analysis (PCA) to exclude genetic outliers and ensure a genetically homogeneous group. Additionally, samples were excluded based on high SNP missingness or abnormal heterozygosity (Data‐Field 22027), chromosomal aneuploidies (Data‐Field 22019) and those that engage in shift work (Data‐Field 826). These samples were identified by UKB (see Bycroft et al. 2018 [14]) and accessible via the data fields reported. After quality control there were 409,630 participants with chronotype data and 239,918 participants with insomnia data.

SNPs were excluded based on imputation quality score, proportion of missing genotypes (< 0.7), minor allele frequency (≤ 0.005), Hardy–Weinberg equilibrium (≤ 1 × 10^−6^), duplication, ambiguity and SNPs missing in the corresponding neuropsychiatric GWAS.

The UKB phenotypes were corrected for the effects of confounders using linear/logistic regression models with chronotype/insomnia as the dependent variable and the confounders as the independent variables. The residuals of these models, representing the variation in phenotype left unexplained by the effects of confounders, were assigned as the corrected phenotypes. The 14 confounders corrected for were: age, sex, UKB assessment centre, genotyping batch and genetic principal components (PCs) 1–10.

### 2.2 Discovery Data for Polygenic Score Analysis—GWAS Summary Statistics

Discovery data for polygenic score analysis included summary statistics from the latest and largest published GWAS of AUT (18,381 cases and 27,969 controls [15]), ADHD (38,691 cases and 186,843 controls [16]), SCZ (74,776 cases and 101,023 controls [17]), BP (41,917 cases and 31,358 controls from a European population [18]) and MDD (170,756 cases and 329,443 controls [19]) (Table S2).

### 2.3 Genome‐Wide Polygenic Score Analysis

Effect sizes for all imputed SNPs were estimated using SBayesRC [20], a method that models SNP effects using a mixture of normal distributions, differing in their variances, and uses SNP annotation information to select the distribution that best models SNP effect sizes. This allows for SNP effect probability distributions to differ across annotation groups and has been shown to perform better than other popular PGS methods. Inputs to SBayesRC include GWAS summary statistics, LD European reference data and 96 functional annotations for the eight million imputed SNPs.

The first step involves converting GWAS summary statistics to COJO format, and converting odds ratios to beta values using log(OR) when beta values were not provided [21]. Subsequently, the SBayesRC tidy function was run to filter SNPs based on inconsistent alleles between the GWAS summary statistic and linkage disequilibrium (LD) data and per SNP sample sizes less than three standard deviations of the mean. The SBayesRC impute function was implemented to impute the GWAS summary data based on LD. This resulted in 7,356,519 SNPs for each phenotype. The main SBayesRC function was then run to estimate SNP effects. These SNP effects were provided to the—score command in Plink1.9 [22] to calculate PGSs for all individuals in the UKB test dataset. Performance metrics were calculated in R by running linear regressions with UKB chronotype or insomnia status as the dependent variable and the SCZ, BP, AUT or ADHD PGS as the independent variables. Variance explained (*R* ^2^) and *p*‐values were obtained using R's summary function. Confidence intervals were computed using the ci_rsquared function as part of the confintr R package (https://github.com/mayer79/confintr).

### 2.4 Pathway‐Based Polygenic Score Analysis

#### 2.4.1 Pathway Data

Pathway data for pathway based PGS analysis was sourced from the file, c2.cp.reactome.v2023.1.Hs.symbols.gmt, from the Human MSigDB Collections curated gene‐sets. The pathways in this file were derived from the Reactome database and filtered to remove inter‐set redundancy by MSigDB. The Reactome database was chosen as it is a peer reviewed database and has a broad representation of biological pathways. To remove very small and overly specialised pathways, we excluded those with less than 10 genes, resulting in 1377 pathways to test.

#### 2.4.2 Pathway‐Based Polygenic Score Analysis Using PRSet

Pathway‐based polygenic score analysis was performed using the tool, PRSet [23]. Inputs to PRSet included Reactome pathway data, published GWAS summary statistics to be used as discovery data, UKB files in PLINK binary format (.bed, .bim and .fam) to be used as target data and a GTF file for the genome build, GRCh37, to map SNPs to genes. SNPs with a reported *p* > 0.5 from the corresponding GWAS were removed. This resulted in final SNP numbers of *N* = 2,374,077 for the AUT PGS, *N* = 2,187,591 for the ADHD PGS, *N* = 2,525,546 for the SCZ PGS and *N* = 2,416,496 for the BP PGS.

Pathway‐based PGS analysis in PRSet involves three steps. The first step is to map SNPs to genes by genomic position. We extended genomic coordinates 35 kb upstream and 10 kb downstream to include potential regulatory elements. The second step is to perform pathway specific LD clumping (parameters used: –clump‐kb 250 kb –clump‐p 1–clump‐r2 0.1). Clumping is performed for each pathway independently to retain the genetic signal for each pathway, that is, to prevent a SNP outside of the pathway being assigned as the lead clumped SNP. The third step involves calculating pathway specific PGSs for each individual, by summing up, for each SNP, the number of minor alleles at that SNP multiplied by the GWAS effect size. Finally, performance metrics (*R* ^2^ and *p*‐value) are calculated for each pathway. Competitive analysis was performed by creating 5000 background polygenic scores, each constructed by randomly sampling the same number of post LD clumped SNPs as contained within gene‐set being tested and conservatively including the gene‐set being tested as one background set. A competitive *p*‐value is computed using a formula that counts the number of times the *p*‐value for a background gene‐set is less than the *p*‐value for the gene‐set being tested and dividing by the number of permutations [23]. A disadvantage of this method is that the lowest possible *p*‐value is 1/(number of permutations +1). Therefore, gene‐sets that reached the lowest possible competitive *p*‐value at 5000 permutations were re‐analysed with 40,000 permutations. Confidence intervals were calculated for the top performing pathways in R using the same method as for the genome‐wide PGSs.

### 2.5 Post Hoc Analysis

#### 2.5.1 Gene and Gene‐Set Enrichment Analyses

Gene‐sets that were statistically significant in the PGS analysis, and the genes within them, were tested for enrichment of common genetic variation using region‐based multi‐marker analysis of genomic annotation (MAGMA; http://ctg.cncr.nl/software/magma [24]) and summary statistics from published GWAS on chronotype (69,369 cases and 236,642 controls of British ancestry [25]) and BP. An analysis involves three steps. First, in the annotation step, SNPs with available GWAS results were mapped on to genes (GRCh37/hg19 start‐stop coordinates +35 kb/−10 kb). Second, in the gene analysis step, gene *p*‐values were computed for each GWAS dataset. This gene analysis is based on a multiple linear principal components regression model that accounts for LD between SNPs in each gene, number of SNPs in each gene, inverse of the mean minor allele count of variants in each gene and the GWAS sample size. The European panel of the 1000 Genomes data was used as a reference panel for LD. Third, a competitive GSA based on the gene *p*‐values, also using a regression structure, was used to test if the genes in each gene‐set were more strongly associated with either phenotype than other genes in the genome.

#### 2.5.2 Co‐Localisation Analysis

We next investigated whether GWAS signals within the two prioritised gene‐sets colocalise with eQTL (expression quantitative trait loci) signals for genes to which the GWAS signals map. To assess colocalisation, we implemented the Bayesian colocalisation test COLOC, defining colocalisation as a high posterior probability that a single variant is responsible for both signals (PP4 > 0.8). This analysis was performed using the coloc R package. eQTL summary data for brain tissues (Amygdala, Hippocampus, Cortex, Frontal Cortex, Anterior Cingulate Cortex, Hypothalamus, Caudate and Nucleus Accumbens) were downloaded from QTLBase v2.0, which aggregates genome‐wide QTL statistics across over 95 tissue and cell types.

#### 2.5.3 eQTL Analysis

Expression quantitative trait loci (eQTL) data was examined using QTLbase2 [26], a database that aggregates genome‐wide QTL summary statistics across 95+ tissue and cell types. We investigated genes within prioritized gene‐sets from pathway‐based PGS analysis that achieved *p* < 1 × 10^−2^ for BP and *p* < 1 × 10^−3^ for chronotype in the gene‐based MAGMA analysis. This identified genes most likely contributing to the significant pathway‐based PGS results. We then examined the most significant SNP at each gene locus in QTLbase to identify associations with expression changes in relevant tissues (brain, nervous system, blood and immune systems). If the top SNP showed no eQTL associations, we explored other SNPs with similarly low *p*‐values. It was assumed that SNPs with the highest statistical significance for BP would also contribute to chronotype prediction, given that BP effect sizes were used to compute the pathway‐based PGSs.

#### 2.5.4 Differential Expression Analysis

Differential expression summary data was downloaded as Table S1 (sheet DGE) from Gandal et al. (2018) [27]. This is a PsychENCODE Consortium study that performed RNA‐sequencing, followed by differential expression analysis of samples isolated form the post‐mortem dorsolateral prefrontal cortex samples for 144 BP cases and 899 controls [27]. We reported false discovery rates (FDR) correcting for all genes tested in the differential expression analysis. To test for a significant difference in the proportions of statistically significant genes in our gene‐sets versus all genes, we used prop.test in R.

An overview of our study design is provided in Figure S1 and more detailed methodology, along with all code used is provided here: https://github.com/laurafahey02/Polygenic_Score_Analyses.

## 3 Results

### 3.1 Genome‐Wide Polygenic Score Analysis

Polygenic scores for AUT (*p* < 2.2 × 10^−16^), BP (*p* = 4.8 × 10^−3^), MDD (*p* = 8.07 × 10^−4^) and SCZ (*p* < 2.2 × 10^−16^) were statistically significant for association with chronotype status. Polygenic scores for ADHD (*p* < 2.2 × 10^−16^), AUT (*p* = 2.93 × 10^−3^), BP (*p* = 2.9 × 10^−7^) and MDD (*p* = < 2.2 × 10^−16^) GWAS phenotypes were statistically significant for association with insomnia, the strongest association being between the ADHD PGS and insomnia. Full statistics are available in Table 1.

**TABLE 1:** Metrics for the performance of genome‐wide polygenic scores based on GWAS effect sizes for ADHD, AUT, BP and SCZ (discovery phenotypes), in the prediction of chronotype and insomnia (target phenotypes).

Discovery phenotype	Target phenotype	*R* ^2^	*R* ^2^ 95% confidence interval: lower	*R* ^2^ 95% confidence interval:Upper	*p*
ADHD	Chronotype	2.25E‐07	0.00E+00	1.14E‐05	7.62E‐01
	Insomnia	2.36E‐03	1.99E‐03	2.76E‐03	< 2.2E‐16
AUT	Chronotype	2.87E‐04	1.92E‐04	4.00E‐04	< 2.2E‐16
	Insomnia	3.69E‐05	4.29E‐06	1.02E‐04	2.93E‐03
BP	Chronotype	1.94E‐05	1.80E‐06	5.58E‐05	4.80E‐03
	Insomnia	1.10E‐04	4.19E‐05	2.09E‐04	2.90E‐07
MDD	Chronotype	2.74E‐05	4.72E‐06	6.88E‐05	8.07E‐04
	Insomnia	2.95E‐03	2.53E‐03	3.39E‐03	< 2.2E‐16
SCZ	Chronotype	2.74E‐05	1.44E‐04	3.28E‐04	< 2.2e‐16
	Insomnia	2.86E‐05	1.75E‐06	8.73E‐05	8.86E‐03

### 3.2 Pathway‐Based Polygenic Score Analysis

**FIGURE 2:**
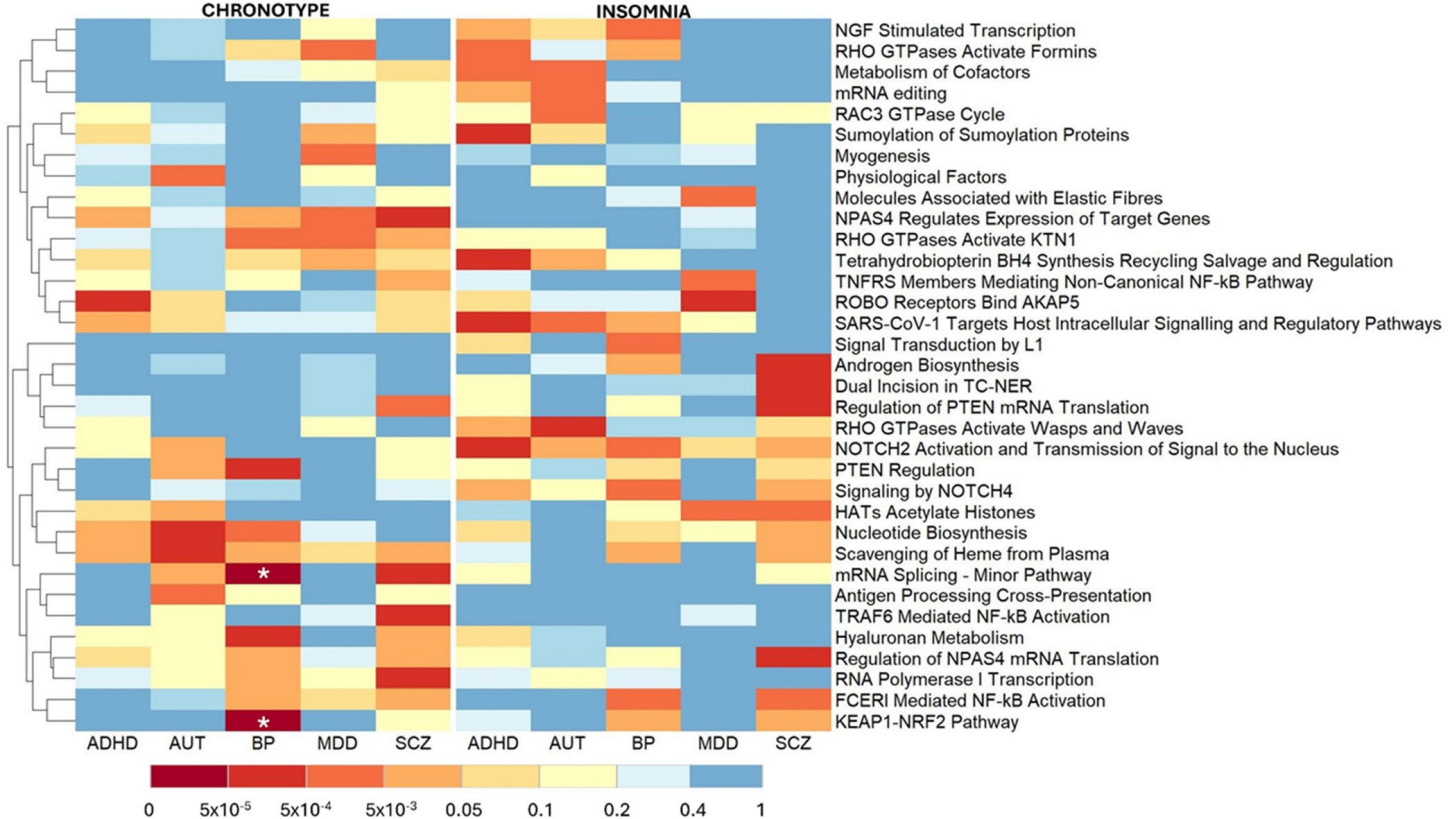
Heatmap of competitive p‐values for the top performing pathways from pathway‐based polygenic score analysis. The rows of this heatmap represent the Reactome pathway‐based PGSs and the columns represent the four NDPC phenotypes. The heatmap is visually separated into two blocks representing pathway‐based PGSs predicting insomnia or chronotype. The top four performing pathway PGSs, out of all 451 tested, for each pair of NDPC and sleep‐related phenotypes are visualised here. Competitive *p*‐values represent the statistical significance of the pathway‐based polygenic scores in predicting insomnia or chronotype, over and above the background genomic signal. The white stars represent pathway based polygenic scores that achieved the lowest possible competitive *p*‐values. The pathway‐based PGSs (rows) are clustered based on similarity of competitive *p*‐values across all eight NDPC—sleep phenotype pairs (columns), as illustrated by the dendrogram.

Two pathways reached the lowest possible competitive *p*‐value based on 40,000 permutations for the prediction of chronotype status based on BP genetic variation: the mRNA Splicing—Minor Pathway (r^2^ = 7.89 × 10^−5^ (CI 3.39 × 10^−5^ to 1.43 × 10^−4^); *p* = 1.3 × 10^−8^; competitive. *p* = 2.5 × 10^−5^) and the KEAP1‐NRF2 pathway (*r* ^2^ = 7.82 × 10^−5^ (CI 3.34 × 10^−5^ to 1.42 × 10^−4^); *p* = 1.52 × 10^−8^; competitive. *p* = 2.5 × 10^−5^) (Figure 2). The mRNA Splicing—Minor Pathway ranked third highest, in terms of competitive *p*‐value, for the prediction of chronotype based on the SCZ PGS (competitive. *p* = 2.5 × 10^−4^) (Figure 2).

### 3.3 Post Hoc Analyses

#### 3.3.1 Interrogations of Significant Pathway‐Based Polygenic Score Analysis Results

**FIGURE 3:**
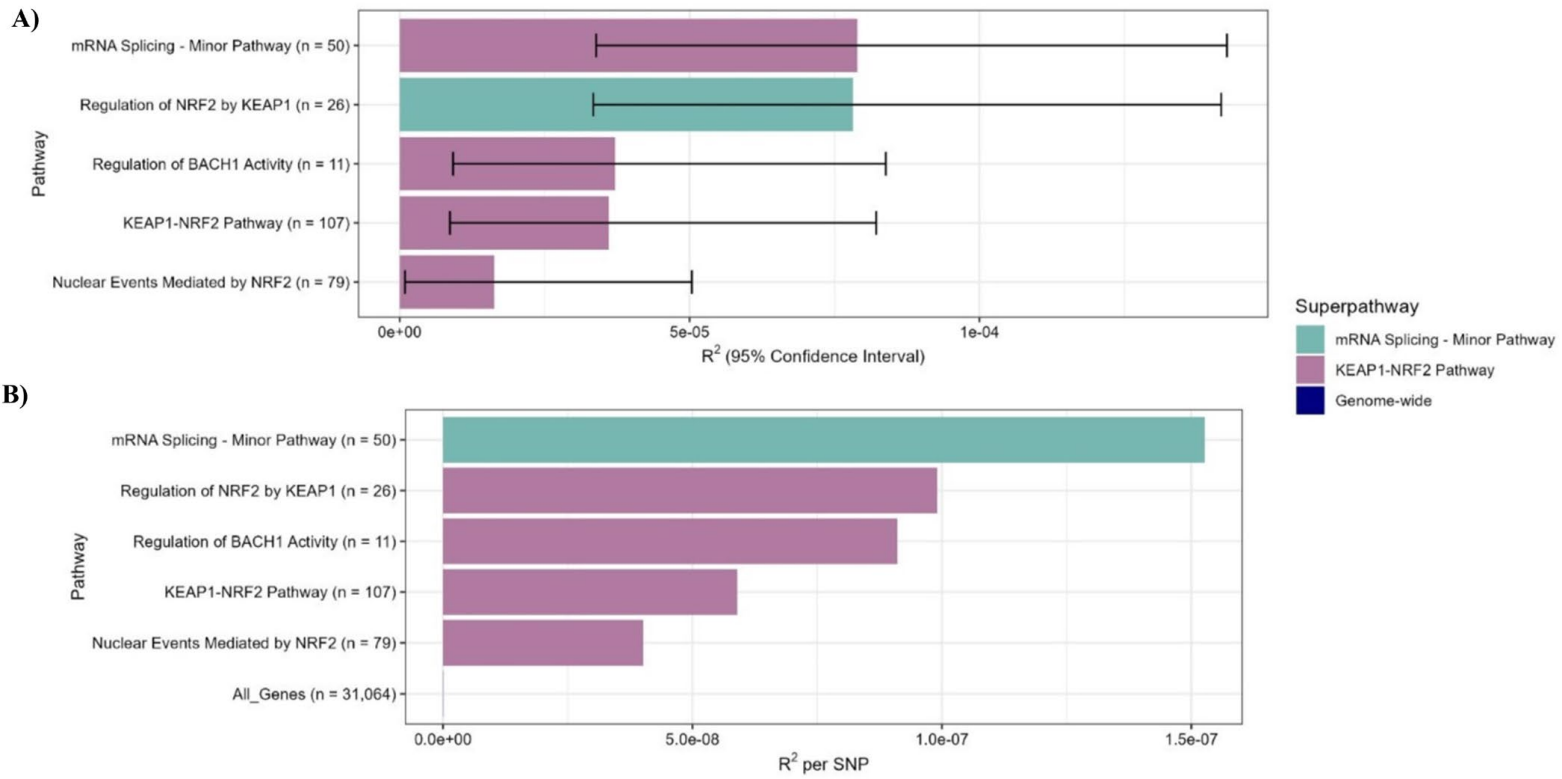
Variance explained (*R* ^2^) by the statistically significant pathway‐based BP PGSs, and their sub‐pathways, in predicting chronotype. (A) Total variance explained per pathway. Horizontal bars show the variance explained (*R* ^2^), error bars indicate 95% confidence intervals. (B) Average variance explained per SNP. This was obtained by dividing the total variance explained per pathway by the number of SNPs in that pathway.

The mRNA Splicing—Minor Pathway encompasses 50 genes, to which 518 SNPs map, while the KEAP1‐NRF2 pathway comprises 107 genes, to which 1356 SNPs map. To explore potential enrichment of the observed signal within specific subsets of these pathways, we investigated Reactome subpathways. Given the relatively small size of the mRNA Splicing—Minor Pathway, it does not contain any Reactome sub‐pathways. The KEAP1‐NRF2 Pathway could be broken down into two Reactome sub‐pathways. We established a third sub‐pathway, Regulation of NRF2 by KEAP1, comprising of all genes that fall outside of the two Reactome sub‐pathways. No KEAP1‐NRF2 sub‐pathway explains more variation than the NRF2‐KEAP1 pathways itself (Figure 3a). To account for the size of the pathways, we plotted the average variance in chronotype explained per SNP for these pathway‐based PGSs, in comparison to the average variance explained per SNP by a PGS constructed using SNPs that map to all HGNC gene symbols (Figure 3b).

#### 3.3.2 Colocalisation Analysis Results

To gain a better understanding of the effect of BP genetic variation on the expression of genes within the KEAP1‐NRF2 and mRNA Splicing—Minor Pathways, we performed colocalisation analysis of eQTL data. Within KEAP1‐NRF2, the gene, *cullin 3* (*CUL3*), achieved PP.H4 > 0.8 in two tissues, the prefrontal cortex and the cortex. This means that there is high probability that BP GWAS signals and eQTL signals at the *CUL3* locus share an underlying casual variant. Each SNP within this locus was also assigned a PP.H4 value, reflecting the posterior probability of each SNP being causal assuming BP GWAS and eQTL signals are shared. The SNP with the highest PP.H4 within *CUL3* was rs4674916 (PP.H4 = 0.99). The eQTL effect size and direction was not available for this SNP. Therefore, we looked at a SNP in perfect LD with this SNP, rs72972378—the BP allele of this SNP is associated with decreased expression of CUL3 in the cortex.

Within the mRNA Splicing—Minor Pathway, the gene*s splicing factor 3b subunit 1* (*SF3B1*), achieved PP.H4 > 0.8 in three tissues, the prefrontal cortex, brain caudate and hippocampus. The SNP with the highest PP.H4 within *SF3B* *1* was rs12621129 (PP.H4 = 0.65 in the hippocampus, PP.H4 = 0.25 in the prefrontal cortex and PP.H4 = 0.16 in the caudate). The BP allele of this SNP is associated with decreased expression of *SF3B1* in the caudate and hippocampus. The effect size and direction was not available for this eQTL in the prefrontal cortex (Table 2).

**TABLE 2:** Colocalisation analysis results.

Gene	PP.H4	SNP	BP allele	BP P	Chronotype P	eQTL allele	Tissue(s)	Effect size	P value	SNP.PP.H4	Expression change associated with BP allele
NRF2‐KEAP1 Pathway											
CUL3	8.73E‐01	rs4674916	C	5.21E‐05	7.40E‐02	NA	Brain‐Prefrontal Cortex and	NA	5.01E‐11	9.99E‐01	NA
	8.95E‐01	rs4674916	C	5.21E‐05	7.40E‐02	NA	Frontal cortex	NA	5.01E‐11	9.99E‐01	NA
	NA	rs72972378 (LD with above SNP = 1)	A	4.54E‐05	7.40E‐02	G	Brain‐Cortex	1.65E‐01	2.52E‐06		Decreased
NRF2‐KEAP1 Pathway											
SF3B1	9.67E‐01	rs12621129	T	5.81E‐06	9.40E‐05	na	Brain‐Prefrontal Cortex	‐4.40E‐02	6.06E‐12	2.64E‐01	NA
	8.36E‐01	rs12621129	T	5.81E‐06	9.40E‐05	C	Brain Caudate	1.75E‐01	5.18E‐05	1.62E‐01	Decreased
	9.45E‐01	rs12621129	T	5.81E‐06	9.40E‐05	C	Brain Hippocampus	9.07E‐02	1.10E‐06	6.54E‐01	Decreased

Abbreviations: PP.H4 = Posterior probability for hypothesis four (both traits are associated and share a single causal variant) of colocalisation analyses; SNP.PP.H4 = Posterior probability of each SNP being causal assuming hypothesis four is true.

#### 3.3.3 Differential Expression Analysis Between BP Cases and Controls in the Post‐Mortem Dorsolateral Prefrontal Cortex

Two genes within the mRNA Splicing—Minor Pathway were differentially expressed: *Serine and Arginine Rich Splicing Factor 6* (*SRSF6*; log2F = −0.07; *p* = 2.0 × 10^−4^; FDR = 0.01) and *Small Nuclear Ribonucleoprotein Polypeptides B and B1* (*SNRPB*; log2FC = 0.08; *p* = 4.4 × 10^−4^, FDR = 0.02). Four differentially expressed genes were observed in the KEAP1‐NRF2 pathway: *Sestrin 1* (*SESN1*; log2FC = 0.07; *p* = 2.8 × 10^−4^; FDR = 0.02), *cyclin dependent kinase inhibitor 1A* (*CDKN1A* log2FC = 0.32; *p* = 2.51 × 10^−4^; FDR = 0.02), *AKT serine/threonine kinase 1* (*AKT1*; log2FC = 0.04; *p* = 6.8 × 10^−4^; FDR = 0.03) and *MAF BZIP Transcription Factor G* (*MAFG*; log2FC = −0.05; *p* = 4.9 × 10^−4^; FDR = 0.02). Interestingly, three of these genes are present in Regulation of NRF2 by KEAP1, which had a higher average *R* ^2^ per SNP than the KEAP1–NRF2 pathway itself (Figure 3b). However, none of these gene‐sets had a statistically significant enrichment of differentially expressed genes (Regulation of NRF2 by KEAP1 *p* = 0.09; KEAP1‐NRF2 *p* = 0.5; mRNA Splicing—Minor Pathway *p* = 0.5).

## 4 Discussion

Using genome‐wide polygenic score analysis, we found polygenic scores for AUT, BP, SCZ and MDD predicted an evening chronotype and polygenic scores for all five NDPC phenotypes predicted insomnia status in UKB. Pathway‐based polygenic score analysis identified the KEAP1‐NRF2 and mRNA Splicing—Minor Pathways as being enriched for genetic variation overlapping between chronotype and BP. The signal was not found to be enriched in any subset of genes within KEAP1–NRF2, but rather seems to be spread across the pathways. Examination of eQTL data using colocalisation analysis identified the *CUL3* gene within KEAP1–NRF2 and the *SF3B1* gene within the mRNA Splicing—Minor Pathway to contain shared causal SNPs associated with both bipolar and expression changes of the respective genes in the brain cortex, caudate and hippocampus. However, genes within these gene‐sets did not show enrichment for differentially expressed genes between BP cases and controls in the dorsolateral prefrontal cortex.

### 4.1 Genome‐Wide Genetic Correlations

Our genome‐wide polygenic score results support previously published genetic correlation findings. AUT [15] and SCZ [25] have been reported to exhibit a negative genetic correlation with a morning chronotype, as demonstrated by linkage disequilibrium score regression. Using a different method and data, our study provides further evidence for these findings, as well as demonstrating a positive association between the BP PGS and an evening chronotype. Additionally, our results confirm that insomnia is genetically correlated with ADHD [16], BP [18], SCZ [28] and MDD [28], the strongest relationship being between ADHD and insomnia. We also report a negative genetic correlation between AUT and insomnia, which was not statistically significant in previous studies [15].

### 4.2 Enrichment of Shared Genetic Variation in Biological Pathways

BP polygenic scores computed using SNPs that map to the KEAP1–NRF2 and mRNA Splicing—Minor Reactome pathways were associated with chronotype, over and above the genome‐wide genetic background signal, indicating shared genetic variation between BP and chronotype to be enriched in these pathways.

For all other phenotype pairs, there was no enrichment of shared genetic variation in any biological pathway, which is somewhat surprising, particularly given the strong genome‐wide correlation between ADHD and insomnia we report. A reason for this could be that the shared genetic variation is highly polygenic, affecting all biological pathways somewhat equally. It could also be that this shared genetic variation is enriched in cell or tissue specific pathways, which we did not explore.

We implemented MAGMA gene‐set analysis to investigate if the KEAP1‐NRF2 and mRNA splicing minor pathways could be identified using standard single phenotype pathway enrichment methods. Neither the KEAP1‐NRF2 nor mRNA splicing minor pathways were enriched for genetic variation individually associated with BP, chronotype or SCZ (Table S3). This indicates that these pathways are enriched for the genetic variation shared between these two phenotypes and are not enriched for the genetic variation associated with each individual phenotype.

#### 4.2.1 The KEAP1–NRF2 Pathway

NRF2 encodes a transcription factor normally bound by KEAP1 in the cytoplasm. It becomes stabilized and translocate to the nucleus under stress conditions, where it binds antioxidant response elements to regulate genes encoding antioxidant and detoxification proteins [29]. NRF2 undergoes rhythmic regulation by BMAL1 and CLOCK [30], and in this way can couple oscillations in redox balance with circadian timekeeping mechanisms.

Elevated oxidative stress has been consistently documented in numerous NDPCs, including BP, and contributes to the concurrent neuroinflammation observed in these conditions [31, 32]. Increased expression of NRF2 target genes help cells mitigate oxidative stress, improve mitochondrial function and reduce inflammation, all involved in NDPC pathology [29, 33]. NRF2 also acts as a negative regulator of the NFκB pro‐inflammatory signalling pathway, while the NFκB pathway reciprocally negatively regulates NRF2 signalling [34]. Given this relationship, it is interesting that the *NRF2* pathways clustered together with the FCERI Mediated NF‐kB Activation Pathway in the competitive *p*‐value heatmap in Figure 2. Given the beneficial roles of NRF2, it may be hypothesized that NRF2 is downregulated in NDPCs. Human postmortem samples from major depressive disorder, SCZ, and BP exhibited significantly lower KEAP1 and NRF2 in the parietal cortex compared to controls [35]. Our eQTL colocalisation results report somewhat the opposite. The gene, *CUL3*, encodes a protein that degrades NRF2; however, the eQTL we found to colocalise with a BP GWAS SNP was in strong LD with a SNP associated with decreased CUL3 expression. Additionally, three of the four genes within the KEAP1‐NRF2 gene‐set that are differentially expressed between BP cases and controls, based on PsychEncode differential expression data, are upregulated.

#### 4.2.2 The mRNA Splicing—Minor Pathway

Splicing of mRNA refers to the removal of introns from pre‐mRNA transcripts, a process performed by the spliceosome. While the majority of splicing events are carried out by the major spliceosome, a subset of introns, known as minor introns, require the minor spliceosome for their excision [36]. This process is less efficient than major splicing, which may contribute to the regulation of genes containing minor introns. Of relevance to NDPCs, the cerebral cortex is one of the tissues with the highest expression of genes containing minor introns [37]. Oscillations in many components of the major spliceosome pathway have been reported to mirror that of *clock* and *bmal1* transcripts [38], indicating that the major spliceosomal pathway is at least partly regulated by the circadian clock. This is interesting in relation to the minor spliceosome too, given the overlap in these pathways. We found the genes, *SF3B1*, which encodes a component of the splicing factor 3b protein complex, to contain a genetic variant causal for BP and associated with decreased *SF3B1* expression. Of the two genes within this gene‐set that are differentially expressed between BP cases and controls in the dorsolateral prefrontal cortex, one is upregulated and the other is downregulated.

### 4.3 Strengths and Limitations

Our study is the first to investigate the biological pathways enriched in genetic variation overlapping between NDPC and sleep phenotypes, using pathway‐based PGS analysis in a global, rather than a hypothesis‐based design. We used the largest available datasets from GWASs of ADHD, AUT, BP as our discovery datasets, and our target dataset consisted of up to 409,630 samples from UKB.

Limitations exist in the pathway‐based PGS method we utilized. First, we mapped SNPs to genes based solely on their physical distance, potentially excluding distal causal genes. While most GWAS SNPs (76%) affect the closest gene [39], some are located in regulatory elements up to hundreds of megabases away from the causal gene. Another limitation is that polygenic score analysis cannot distinguish between horizontal versus vertical pleiotropy, that is, whether genetic variation within the KEAP1–NRF2 and mRNA splicing minor pathways independently contribute to both BP and an evening chronotype, or whether genetic variation within these pathways contribute to an evening chronotype, which in turn, causes BP, or vice versa. Another limitation is that the number of insomnia samples (239,918) was less than the number of chronotype samples (409,630). A final limitation is that due to the computational intensity of the permutation analysis, we could only test gene sets from a single pathway database, Reactome. Different results might be obtained using pathway definitions from other databases, such as Gene Ontology or Biocarta.

Neither the KEAP1‐NRF2 nor the mRNA‐splicing minor pathways were found to be enriched in genes differentially expressed in the *post‐mortem* dorsolateral prefrontal cortex of BP patients. A limitation of this analysis is that only one specific brain region was investigated. It is possible that these genes are differentially expressed in a different brain region.

### 4.4 Implications and Future Analyses

Our study provides biological insight into the breakdown of genomic correlations between NDPC and sleep phenotypes across 1377 biological pathways. It must be noted that the *R* ^2^ values of the significant pathway‐based PGSs are low. This is partly to be expected, given the small numbers of genes in these pathways. Clear enrichment of explained variance by the mRNA Splicing—Minor and KEAP1‐NRF2 pathways are observed when plotting the average variance explained per SNP for these pathways, in comparison to the average variance explained per SNP by a PGS computed using all genes (Figure 3b).

Future analyses could further investigate the disturbance of these pathways in BP patients with circadian dysfunction. A genomic analysis could select BP cases with high KEAP1‐NRF2/mRNA‐splicing minor PGSs and perform a differential expression analysis on just these cases in biologically relevant tissues (this would require genotype and RNA‐sequencing data on BP cases and controls).

## 5 Conclusion

Our findings provide further evidence of shared genetic variation between NDPC and sleep traits and highlight the KEAP1‐NRF2 and mRNA Splicing—Minor pathways as being enriched in shared genetic variation between chronotype and BP. Both of these pathways have previously been shown to be at least partially regulated by the circadian clock, which provides further support the hypothesis that disrupted circadian rhythm and seasonal adaptive responses underlie the pathology of BP. Further research is necessary to determine the clinical feasibility of targeting these pathways for intervention.

## Conflicts of Interest

The authors declare no conflicts of interest.

## Supporting information


**Data S1.** Supporting Information.

## Data Availability

The data that supports the findings of this study are available in the Supporting Information of this article.

## References

[1] American Psychiatric Association (APA) , Diagnostic and Statistical Manual of Mental Disorders (DSM‐V) 5th ed. (Washington DC: American Psychiatric Publishing, 2013).

[2] S. Cohrs , “Sleep Disturbances in Patients With Schizophrenia,” CNS Drugs 22, no. 11 (2008): 939–962. 18840034 10.2165/00023210-200822110-00004

[3] F. Cortesi , F. Giannotti , A. Ivanenko , and K. Johnson , “Sleep in Children With Autistic Spectrum Disorder,” Sleep Medicine 11, no. 7 (2010): 659–664. 20605110 10.1016/j.sleep.2010.01.010

[4] J. Galli , E. Loi , L. M. Visconti , et al., “Sleep Disturbances in Children Affected by Autism Spectrum Disorder,” Frontiers in Psychiatry 17 (2022): 13. 10.3389/fpsyt.2022.736696 PMC8891952 35250655

[5] D. Wajszilber , J. A. Santisteban , and R. Gruber , “Sleep Disorders in Patients With ADHD: Impact and Management Challenges,” Nature and Science of Sleep 10 (2018): 453–480. 10.2147/NSS.S163074 PMC6299464 30588139

[6] M. M. Ohayon and T. Roth , “Place of Chronic Insomnia in the Course of Depressive and Anxiety Disorders,” Journal of Psychiatric Research 37, no. 1 (2003): 9–15. 12482465 10.1016/s0022-3956(02)00052-3

[7] M. Von Schantz , M. A. Leocadio‐Miguel , M. J. McCarthy , S. Papiol , and D. Landgraf , “Genomic Perspectives on the Circadian Clock Hypothesis of Psychiatric Disorders,” Advances in Genetics 107 (2021): 153–191. 33641746 10.1016/bs.adgen.2020.11.005

[8] M. Von Schantz , “Natural Variation in Human Clocks,” Advances in Genetics 99 (2017): 73–96. 29050555 10.1016/bs.adgen.2017.09.003

[9] H. Zou , H. Zhou , R. Yan , Z. Yao , and Q. Lu , “Chronotype, Circadian Rhythm, and Psychiatric Disorders: Recent Evidence and Potential Mechanisms,” Frontiers in Neuroscience 16 (2022): 811771. 36033630 10.3389/fnins.2022.811771 PMC9399511

[10] S. Mistry , J. R. Harrison , D. J. Smith , V. Escott‐Price , and S. Zammit , “The Use of Polygenic Risk Scores to Identify Phenotypes Associated With Genetic Risk of Schizophrenia: Systematic Review,” Schizophrenia Research 197 (2018): 2–8. 29129507 10.1016/j.schres.2017.10.037

[11] K. Ohi , R. Ochi , Y. Noda , et al., “Polygenic Risk Scores for Major Psychiatric and Neurodevelopmental Disorders Contribute to Sleep Disturbance in Childhood: Adolescent Brain Cognitive Development (ABCD) Study,” Translational Psychiatry 11, no. 1 (2021): 187. 33771979 10.1038/s41398-021-01308-8 PMC7997961

[12] M. X. Carpena , C. Bonilla , A. Matijasevich , et al., “Sleep‐Related Traits and Attention‐Deficit/Hyperactivity Disorder Comorbidity: Shared Genetic Risk Factors, Molecular Mechanisms, and Causal Effects,” World Journal of Biological Psychiatry 22, no. 10 (2021): 778–791. 10.1080/15622975.2021.1907719 33821771

[13] K. S. O'Connell , O. Frei , S. Bahrami , et al., “Characterizing the Genetic Overlap Between Psychiatric Disorders and Sleep‐Related Phenotypes,” Biological Psychiatry 90, no. 9 (2021): 621–631. 34482950 10.1016/j.biopsych.2021.07.007

[14] C. Bycroft , C. Freeman , D. Petkova , et al., “The UK Biobank Resource With Deep Phenotyping and Genomic Data,” Nature 562, no. 7726 (2018): 203–209. 30305743 10.1038/s41586-018-0579-z PMC6786975

[15] J. Grove , S. Ripke , T. D. Als , et al., “Identification of Common Genetic Risk Variants for Autism Spectrum Disorder,” Nature Genetics 51, no. 3 (2019): 431–444. 30804558 10.1038/s41588-019-0344-8 PMC6454898

[16] D. Demontis , R. K. Walters , J. Martin , et al., “Discovery of the First Genome‐Wide Significant Risk Loci for Attention Deficit/Hyperactivity Disorder,” Nature Genetics 51, no. 1 (2019): 63–75. 30478444 10.1038/s41588-018-0269-7 PMC6481311

[17] V. Trubetskoy , A. F. Pardiñas , T. Qi , et al., “Mapping Genomic Loci Implicates Genes and Synaptic Biology in Schizophrenia,” Nature 604, no. 7906 (2022): 502–508. 35396580 10.1038/s41586-022-04434-5 PMC9392466

[18] N. Mullins , A. J. Forstner , K. S. O'Connell , et al., “Genome‐Wide Association Study of More Than 40,000 Bipolar Disorder Cases Provides New Insights Into the Underlying Biology,” Nature Genetics 53, no. 6 (2021): 817–829. 34002096 10.1038/s41588-021-00857-4 PMC8192451

[19] N. R. Wray , S. Ripke , M. Mattheisen , et al., “Genome‐Wide Association Analyses Identify 44 Risk Variants and Refine the Genetic Architecture of Major Depression,” Nature Genetics 50, no. 5 (2018): 668–681. 29700475 10.1038/s41588-018-0090-3 PMC5934326

[20] Z. Zheng , S. Liu , J. Sidorenko , et al., “Leveraging Functional Genomic Annotations and Genome Coverage to Improve Polygenic Prediction of Complex Traits Within and Between Ancestries,” Nature Genetics 56, no. 5 (2024): 767–777. 38689000 10.1038/s41588-024-01704-y PMC11096109

[21] M. Sogl , D. Taeger , D. Pallapies , et al., “Quantitative Relationship Between Silica Exposure and Lung Cancer Mortality in German Uranium Miners, 1946–2003,” British Journal of Cancer 107, no. 7 (2012): 1188–1194. 22929885 10.1038/bjc.2012.374 PMC3461166

[22] S. Purcell , B. Neale , K. Todd‐Brown , et al., “PLINK: A Tool Set for Whole‐Genome Association and Population‐Based Linkage Analyses,” American Journal of Human Genetics 81, no. 3 (2007): 559–575. 17701901 10.1086/519795 PMC1950838

[23] S. W. Choi , J. García‐González , Y. Ruan , et al., “PRSet: Pathway‐Based Polygenic Risk Score Analyses and Software,” PLoS Genetics 19, no. 2 (2023): e1010624. 36749789 10.1371/journal.pgen.1010624 PMC9937466

[24] C. A. De Leeuw , J. M. Mooij , T. Heskes , and D. Posthuma , “MAGMA: Generalized Gene‐Set Analysis of GWAS Data,” PLoS Computational Biology 11, no. 4 (2015): e1004219. 25885710 10.1371/journal.pcbi.1004219 PMC4401657

[25] S. E. Jones , J. M. Lane , A. R. Wood , et al., “Genome‐Wide Association Analyses of Chronotype in 697,828 Individuals Provides Insights Into Circadian Rhythms,” Nature Communications 10, no. 1 (2019): 343. 10.1038/s41467-018-08259-7 PMC6351539 30696823

[26] Z. Zheng , D. Huang , J. Wang , et al., “QTLbase: An Integrative Resource for Quantitative Trait Loci Across Multiple Human Molecular Phenotypes,” Nucleic Acids Research 48, no. D1 (2020): D983–D991. 31598699 10.1093/nar/gkz888 PMC6943073

[27] M. J. Gandal , P. Zhang , E. Hadjimichael , et al., “Transcriptome‐Wide Isoform‐Level Dysregulation in ASD, Schizophrenia, and Bipolar Disorder,” Science 362, no. 6420 (2018): eaat8127. 30545856 10.1126/science.aat8127 PMC6443102

[28] K. Watanabe , P. R. Jansen , J. E. Savage , et al., “Genome‐Wide Meta‐Analysis of Insomnia Prioritizes Genes Associated With Metabolic and Psychiatric Pathways,” Nature Genetics 54, no. 8 (2022): 1125–1132. 35835914 10.1038/s41588-022-01124-w

[29] R. Bhandari , J. Kaur , S. Kaur , and A. Kuhad , “The Nrf2 Pathway in Psychiatric Disorders: Pathophysiological Role and Potential Targeting,” Expert Opinion on Therapeutic Targets 25, no. 2 (2021): 115–139. 33557652 10.1080/14728222.2021.1887141

[30] J. O. Early , D. Menon , C. A. Wyse , et al., “Circadian Clock Protein BMAL1 Regulates IL‐1β in Macrophages via NRF2,” Proceedings of the National Academy of Sciences of the United States of America 115, no. 36 (2018): E8460–E8468. 30127006 10.1073/pnas.1800431115 PMC6130388

[31] S. Salim , “Oxidative Stress and Psychological Disorders,” Current Neuropharmacology 12, no. 2 (2014): 140–147. 24669208 10.2174/1570159X11666131120230309 PMC3964745

[32] D. M. Teleanu , A. G. Niculescu , I. I. Lungu , et al., “An Overview of Oxidative Stress, Neuroinflammation, and Neurodegenerative Diseases,” International Journal of Molecular Sciences 23, no. 11 (2022): 5938. 35682615 10.3390/ijms23115938 PMC9180653

[33] G. Morris , A. J. Walker , K. Walder , et al., “Increasing Nrf2 Activity as a Treatment Approach in Neuropsychiatry,” Molecular Neurobiology 58, no. 5 (2021): 2158–2182. 33411248 10.1007/s12035-020-02212-w

[34] W. Gao , L. Guo , Y. Yang , et al., “Dissecting the Crosstalk Between Nrf2 and NF‐κB Response Pathways in Drug‐Induced Toxicity,” Frontiers in Cell and Development Biology 2 (2022): 9. 10.3389/fcell.2021.809952 PMC8847224 35186957

[35] K. Hashimoto , “Essential Role of Keap1‐Nrf2 Signaling in Mood Disorders: Overview and Future Perspective,” Frontiers in Pharmacology 9 (2018): 1182. 30386243 10.3389/fphar.2018.01182 PMC6198170

[36] E. El Marabti , J. Malek , and I. Younis , “Minor Intron Splicing From Basic Science to Disease,” International Journal of Molecular Sciences 22, no. 11 (2021): 6062. 34199764 10.3390/ijms22116062 PMC8199999

[37] A. M. Olthof , K. C. Hyatt , and R. N. Kanadia , “Minor Intron Splicing Revisited: Identification of New Minor Intron‐Containing Genes and Tissue‐Dependent Retention and Alternative Splicing of Minor Introns,” BMC Genomics 20, no. 1 (2019): 686. 31470809 10.1186/s12864-019-6046-x PMC6717393

[38] S. M. Buel , S. Debopadhaya , H. De los Santos , et al., “The PAICE Suite Reveals Circadian Posttranscriptional Timing of Noncoding RNAs and Spliceosome Components in *Mus musculus* Macrophages,” G3 Genes|Genomes|Genetics 12, no. 9 (2022): jkac176. 35876788 10.1093/g3journal/jkac176 PMC9434326

[39] J. Nasser , D. T. Bergman , C. P. Fulco , et al., “Genome‐Wide Enhancer Maps Link Risk Variants to Disease Genes,” Nature 593, no. 7858 (2021): 238–243. 33828297 10.1038/s41586-021-03446-x PMC9153265

[40] H. S. Dashti , S. E. Jones , A. R. Wood , et al., “Genome‐Wide Association Study Identifies Genetic Loci for Self‐Reported Habitual Sleep Duration Supported by Accelerometer‐Derived Estimates,” Nature Communications 10, no. 1 (2019): 1100. 10.1038/s41467-019-08917-4 PMC6405943 30846698

[41] H. Wang , J. M. Lane , S. E. Jones , et al., “Genome‐Wide Association Analysis of Self‐Reported Daytime Sleepiness Identifies 42 Loci That Suggest Biological Subtypes,” Nature Communications 10, no. 1 (2019): 3503. 10.1038/s41467-019-11456-7 PMC6692391 31409809

