# Supplementary material for: Shared Genetic Links Between Sleep, Neurodevelopmental and Neuropsychiatric Conditions: A Genome‐Wide and Pathway‐Based Polygenic Score Analysis
Source: Genes Brain Behav. 2024 Dec 26;23(6):e70011. doi: 10.1111/gbb.70011 (PMC11669943; doi:10.1111/gbb.70011)
Supplement: Supplementary file 1 — Data S1. Supporting Information. [file GBB-23-e70011-s001.docx]

Supplementary Information

Shared Genetic Links between Sleep, Neurodevelopmental and Neuropsychiatric Conditions: A Genome-Wide and Pathway-Based Polygenic Score Analysis

Laura Fahey, Lorna M. Lopez

# Table of Contents

| **Name** | **Page Number** | **Description** |
| --- | --- | --- |
| [Supplementary Figure 1](#fig1) | 2 | Overview of study design. |
| [Supplementary Table 1](#table1) | 3 | Encoding of the chronotype and insomnia questionnaire phenotypes from UK Biobank. |
| [Supplementary Table 2](#table2) | 4 | GWAS summary statistic data using as discovery data for polygenic score analysis |
| [Supplementary Table 3](#table3) | 5 | MAGMA gene-set analysis results |


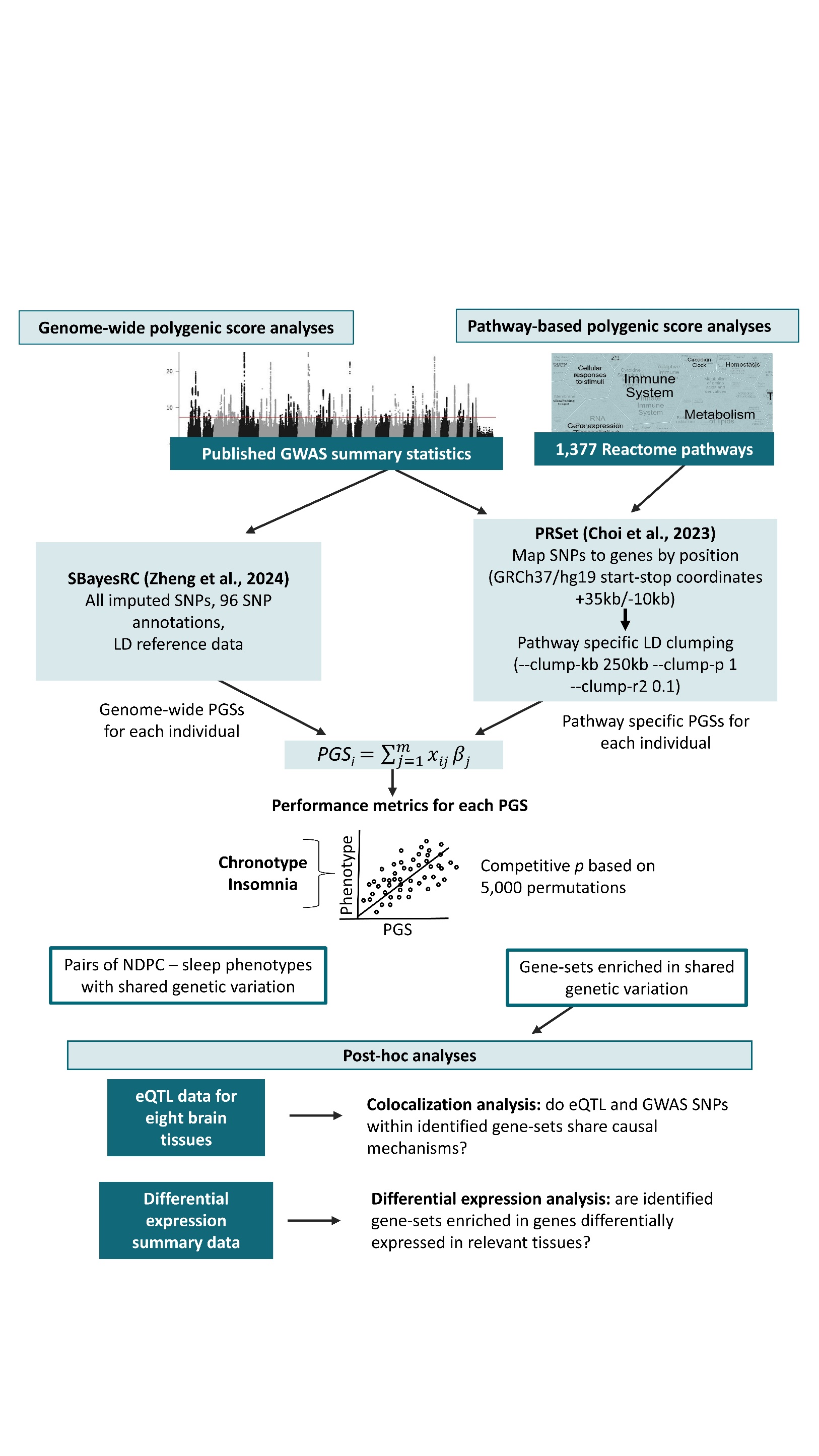


**Supplementary Figure 1: Overview of study design.**

**Supplementary Table 1: Encoding of the chronotype and insomnia questionnaire phenotypes from UK Biobank.**

| **Phenotype** | **Questions** | **Answers** | **Encoding** | **Number of participants included after quality control** |
| --- | --- | --- | --- | --- |
| **Chronotype** | Answer to the question, “do you consider yourself to be”? | Definitely an ‘evening’ person | 4 | 409,630 |
|  |  | More an ‘evening’ than a ‘morning’ person | 3 |  |
|  |  | Do not know | Missing |  |
|  |  | More a ‘morning’ than ‘evening’ person | 2 |  |
|  |  | Definitely a ‘morning’ person | 1 |  |
| **Insomnia** | Answer to the question, “Do you have trouble falling asleep at night or do you wake up in the middle of the night?” | never/rarely | 0 | 239,918 |
|  |  | sometimes | Missing |  |
|  |  | usually | 1 |  |
|  |  | Prefer not to answer | Missing |  |

**Supplementary Table 2: GWAS summary statistic data using as discovery data for polygenic score analysis**

| **Phenotype** | **Sample size** | **Population** | **Reference** | **File used** |
| --- | --- | --- | --- | --- |
| ADHD | 38,691 cases and 186,843 controls | European, Danish and Icelandic populations | Demontis et al. (2019) | <https://figshare.com/articles/dataset/adhd2022/22564390/ADHD_meta_Jan2022_iPSYCH1_iPSYCH2_deCODE_PGC.gz> |
| Autism | 18,381 cases and 27,969 controls | Danish | Grove et al. (2019) | <https://figshare.com/articles/dataset/asd2019/14671989/iPSYCH-PGC_ASD_Nov2017.gz> |
| Bipolar Disorder | 40,463 cases and 313,436 controls | European | Mullins et al. (2021) | <https://figshare.com/articles/dataset/bip2021_noUKBB/22564402/daner_bip_pgc3_nm_noukbiobank.gz> |
| Schizophrenia | 67,323 cases and 93,456 controls | European, East Asian, African American and Latino | Trubetskoy et al. (2019) | <https://figshare.com/articles/dataset/scz2022/19426775/daner_PGC_SCZ_w3_90_0418b_ukbbdedupe> |
| Major Depressive Disorder | 170,756 cases and 329,443 controls | European | Wray et al. (2018) | <https://datashare.ed.ac.uk/bitstream/handle/10283/3203/PGC_UKB_depression_genome-wide.txt> |

**Supplementary Table 3: MAGMA gene-set analysis results.**

| **GWAS Phenotype** | **Gene-set** | **N Genes** | **Beta** | **Standard Error** | **P Value** |
| --- | --- | --- | --- | --- | --- |
| BP | mRNA splicing - minor pathway | 49 | -0.17384 | 0.16224 | 0.85801 |
|  | KEAP1 NRF2 Pathway | 104 | 0.061078 | 0.10114 | 0.27296 |
|  |  |  |  |  |  |
| Chronotype | mRNA splicing - minor pathway | 49 | 0.14526 | 0.10701 | 0.087337 |
|  | KEAP1 NRF2 Pathway | 104 | 0.057203 | 0.10244 | 0.28829 |
|  |  |  |  |  |  |
| SCZ | mRNA splicing - minor pathway | 49 | 0.097795 | 0.18121 | 0.29471 |
|  | KEAP1 NRF2 Pathway | 104 | 0.1943 | 0.10769 | 0.035601 |

Each gene-set was tested for enrichment of genome-wide association study (GWAS) identified genetic variation associated with bipolar disorder (BP), chronotype and schizophrenia (SCZ).
